# 3-Nitro-4-(tetrazol-5-yl) furazan: theoretical calculations, synthesis and performance†

**DOI:** 10.1039/c8ra02682c

**Published:** 2018-04-18

**Authors:** Zhiyue Han, Qi Jiang, Zhiming Du, Yupeng Zhang, Yuezhen Yang

**Affiliations:** State Key Laboratory of Explosion Science and Technology, Beijing Institute of Technology 5 South Zhongguancun Street, Haidian District Beijing 100081 China hanzhiyue@bit.edu.cn +86-01-68912765 +86-01-68912765

## Abstract

The synthesis mechanism of 3-nitro-4-(tetrazol-5-yl)furazan (NTZF) was calculated by Gaussian 09 for the first time, and NTZF was successfully synthesized based on the theoretical design. Its ionic salts (RbNTZF and CsNTZF) were synthesized and studied by single-crystal X-ray diffraction firstly. The thermal stability of NTZF was investigated by TG-DSC and the kinetic data of thermal decomposition were calculated. The sensitivity of NTZF was measured. The formation heat, detonation velocity (*D*) and detonation pressure (*P*) of NTZF were calculated. NTZF is insensitive to impact and friction (impact > 40 J, friction > 360 J) and has higher detonation velocity and pressure (*D* = 7.838 km s^−1^, *P* = 27.32 GPa) compared to TNT (*D* = 6881 m s^−1^, *P* = 19.5 GPa). NTZF has appropriate sensitivity and detonation performance, so it can be used as a low explosive and gas generant.

## Abstract 2

The synthesis mechanism of 3-nitro-4-(tetrazol-5-yl)furazan (NTZF) was calculated by Gaussian 09 for the first time, and NTZF was successfully synthesized based on the theoretical design.

## Graphical abstract

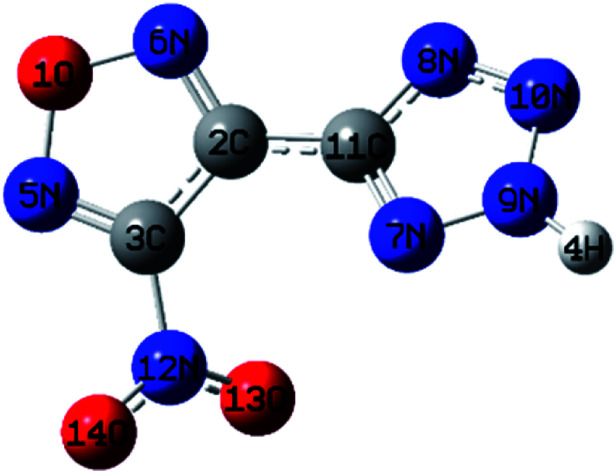

## 1. Introduction

As a new type of energetic materials, nitrogen-rich compounds contain a high enthalpy of formation and high energy density. Their molecular structures include a large number of chemical bonds N–N, C–N, N=N, C–N. Some energetic nitrogen-rich compounds have been synthesized for application in the field of military chemistry and explosion safety, such as high-energy insensitive explosives,^1,2^ small propulsion system solid fuels,^3,4^ smokeless pyrotechnics,^5,6^ gas generators,^7,8^ flame low temperature fire extinguishing agents^9,10^ and so on.

The molecular formula of NTZF is C_3_N_7_HO_3_. The nitrogen content of it is 53.55%. The structure of it is very stable because of the π-conjugate formed by the heterocyclic system. Thus, the NTZF is a stable energetic compound which has good application prospects in the field of explosives. NTZF was synthesized for the first time by Wang^11^ with ATZF as the raw material. However, the synthesis mechanism of NTZF hasn't been studied to date.

In this study, the synthesis mechanism of NTZF was studied by theoretical calculations for the first time, and its structure was characterized by IR, ^1^H NMR, ^13^C NMR and EA. Single crystals of its ionic salts were cultivated firstly. The thermal stability was investigated by TG-DSC and friction sensitivity was measured. Additionally, its formation heat, detonation pressure and detonation velocity were calculated.

## 2. Experimental section

### 2.1 Synthesis of 3-nitro-4-(tetrazol-5-yl)furazan (NTZF)

All chemical used reagents and solvents were analytically pure, and purchased commercially.

In this paper, 3-amino-4-(tetrazol-5-yl)furazan (ATZF) was synthesized according to the method in ref. 12. NTZF was synthesized according to the theoretical optimization results:

Concentrated H_2_SO_4_ (4 mL) and ATZF (0.76 g, 5 mmol) were added to a three-necked flask at room temperature. The reaction mixture was stirred until the solid had dissolved. Na_2_WO_4_ (2.5 g, 7.5 mmol) was added to 30% H_2_O_2_ (24 mL) and the mixture was stirred gently at −10 °C, until the solid had completely dissolved. The solution was added dropwise to the three-necked flask where a vigorous exothermic reaction occurred. The temperature of the reaction was adjusted to 35 °C and keep 3 h. The resulting solution was filtered and extracted with acetate (20 mL × 3). The yellow-green product of NTZF were washed with saturated brine, dried and evaporated. Yield 77.50%. C_3_N_7_HO_3_: calcd C 20.04, N 52.12, H 0.87%; found: C 19.68, N 53.55, H 0.55%; IR (KBr, *ν* cm^−1^): 1566.77, 1328.01 (–NO_2_), 3095.65, 1144.77, 1114.42 (Tetrazole ring), 1471.58, 1397.51, 996.10 (Furan ring). ^1^H NMR (DMSO-d_6_, *δ*/ppm): 12.17 (s, 1H). ^13^C NMR (DMSO-d_6_, *δ*/ppm): 160.02, 147.37, 141.34.

RbNTZF and CsNTZF: the synthesis of RbNTZF/CsNTZF was carried out from NTZF by reaction with rubidium/cesium. 1.83 g (10 mmol) NTZF was added in a three-necked flask with 20 mL of water at 40 °C. After stirred fully to dissolve, the mixture was added 1.15 g (5 mmol) rubidium carbonate/1.63 g (5 mmol) cesium carbonate. The reaction produced a large number of bubbles. After fully responsed to clarification, the reaction liquid was filtered and extracted. The filtrate was mixed with toluene by certain proportion. The product was steamed at 60 °C and dried backspin steamed.

### 2.2 Single crystal cultivation

A portion of the solution extracted during the above reaction was removed. The sample was placed in a clean beaker after filtering. The beaker was placed in a thermostatic incubator for 3 days until the colorless crystal which can be used for single crystal test appear.

### 2.3 Measurements

Infrared spectra (IR) was recorded with a Nicolet FT-IR6700. Elemental analysis was measured with a Vario EL elemental analyzer. The NMR spectrum was carried out with a DRX500 Bruker NMR spectrometer. Single crystal structure characterization was determined by Rigaku 742+ single crystals diffractometer.

Differential scanning calorimeter (DSC) and thermogravimetric analysis (TG) were carried out with a STARe system and a CDR-4P differential scanning calorimeter and thermogravimetric analyzer. TG-DSC of ATZF was measured under nitrogen with a 50 mL min^−1^ flux over the temperature ramp of 50–400 °C, at a heating rate of 2, 5, 10, 20 °C min^−1^.

The impact and the friction sensitivities were tested according to BAM standard methods. The flame sensitivity was determined by HGY-1 flame sensitivity instrument. The sample mass used for each test was 20 ± 2 mg. The sample was compressed into a fire cap at 58.8 MPa and the cap was ignited by the standard black powder column. The 50% ignition height indicated the friction sensitivity.

## 3. Result and discussion

### 3.1 Theoretical calculation

#### 3.1.1 Structure of NTZF

**Fig. 1:**
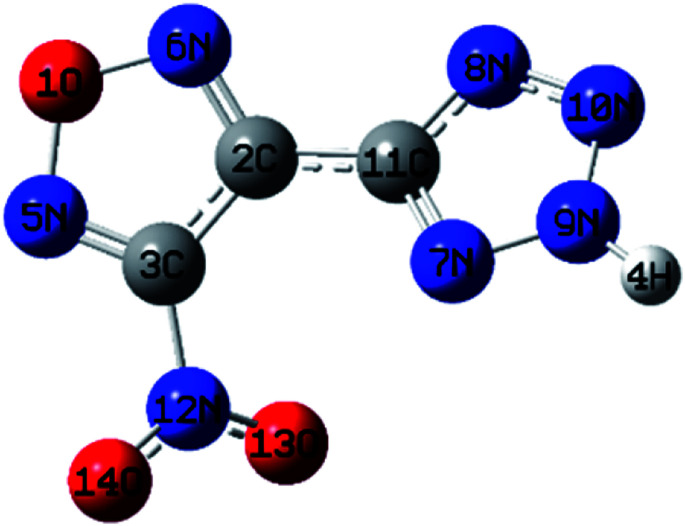
Geometry of NTZF.

The structure of NTZF was optimized with B3LYP/6-311 ++ G ** (ref. 13) using the Gaussian 09 software package.^14^ The geometric structure of the NTZF molecule is shown in Fig. 1 and the bond lengths and bond angles are shown in ESI Tables 1 and 2.†

Based on the principle of minimum energy, the lone pair electrons on one oxygen atom of the nitro group repulsively opposes the lone pair of nitrogen atoms in tetrazole. The furazan ring, tetrazole ring and nitro in NTZF are not coplanar. The bond length of furazan ring is between 1.30–1.43 Å and the bond angles are similar to each other. Therefore, it tends to form a stable five-membered ring structure. The repulsion between the lone pair of the oxygen atom and nitrogen atoms makes the bond angles of the N5–O1–N4 in the furazan ring become larger. What's more, the bond length in the tetrazole ring is between 1.30–1.36 Å and the bond angles are similar. It is also a stable five-membered ring structure. In summary, the optimized molecular structure of NTZF is stable.

#### 3.1.2 NBO

Natural bond orbital (NBO) analysis was performed on the geometry of the optimized NTZF at B3LYP/6-311 ++ G ** level. The NBO charge distribution of each atom in NTZF was obtained (Table 1). The heavy electron attracting effect of nitro in NTZF makes the C3 atom positively charged. N12 atom is also positively charged due to the electronegativity in oxygen is greater than that in nitrogen. The electronegativity of the oxygen in the furazan ring is larger and affected by electrons in the 4 and 5 positions. Therefore, the negative charge in N5 and N6 of the ring is less. The electron-withdrawing ability of nitro in Π^3^_4_ system is larger than that in π-conjugated tetrazole, therefore, the positive charge carried by C3 is much larger than C2. The conjugate system of the tetrazole ring accepts the electron the hydrogen atom was supplied with, so that the hydrogen with the positive charge is more easily ionized.

**Table 1:** NBO charge of NTZF

Atom	Charge/a.u.	Atom	Charge/a.u.
O1	−0.11	N8	−0.23
C2	0.07	N9	−0.13
C3	0.23	N10	−0.03
H4	0.42	C11	0.25
N5	−0.05	N12	0.46
N6	−0.03	O13	−0.31
N7	−0.23	O14	−0.30

#### 3.1.3 ESP

**Fig. 2:**
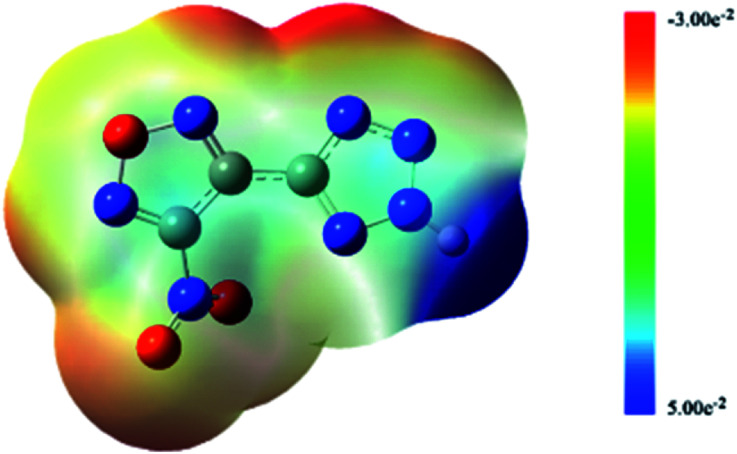
ESPs of NTZF.

Fig. 2 shows the ESPs for the 0.001 electronic per bohr^3^ isosurfaces of electron density elaluated at the B3LYP/6-311++G** level. The colors range from −3.00 × 10^−2^ to +5.00 × 10^−2^ eV with dark blue denoting extremely electron-deficient regions [*V*(*r*) ≥ 5.00 × 10^−2^] and red denoting electron-rich regions [*V*(*r*) ≤ −3.00 × 10^−2^].

As shown in Fig. 2, the hydrogen atom on the tetrazole ring can easily lose, because the positive potential of it is the maximum. It shows that the NTZF can become an anion. The nitro and oxygen atom of furazan, nitrogen atom of tetrazole and nitro group are negative potential distribution. Among them, the negative potential of the nitroxide, the nitrogen atom of the furazan ring and part of the nitrogen atoms on the tetrazole ring are close to the maximum. Therefore, these atoms all have coordination ability. The results of ESP of NTZF are consistent with the results of NBO.

#### 3.1.4 HOMO–LOMO

**Fig. 3:**
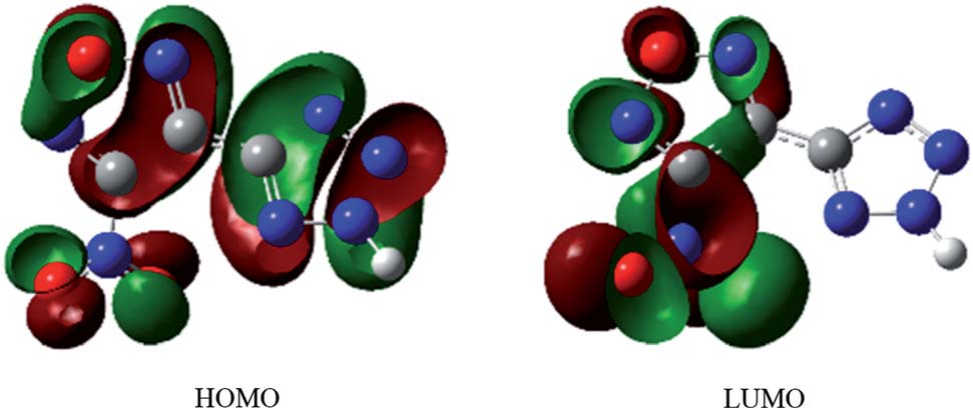
The HOMO, LUMO orbitals of NTZF.

Fig. 3 shows the HOMO–LUMO orbitals of the NTZF based on the molecular frontier orbital theory. When the electrons in NTZF are stimulated by external energy, the electrons in the tetrazolyl will transfer toward the furazan ring and the nitro group. The energy of the HOMO orbitals of the NTZF is −9.07 eV and the energy of the LUMO orbitals is −3.66 eV. The difference between the two molecular orbitals (Δ*E* = 5.42 eV) is the energy required for the electrons in the molecule to excite from the ground state to the excited state. Above all, the molecule is relatively stable.

### 3.2 Synthesis mechanism of NTZF

**Fig. 4:**
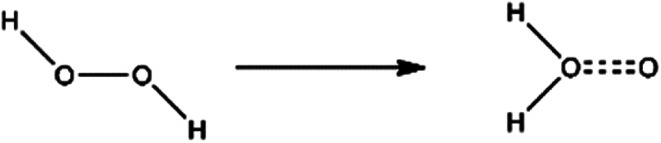
Isomerization of H_2_O_2_.

**Fig. 5:**
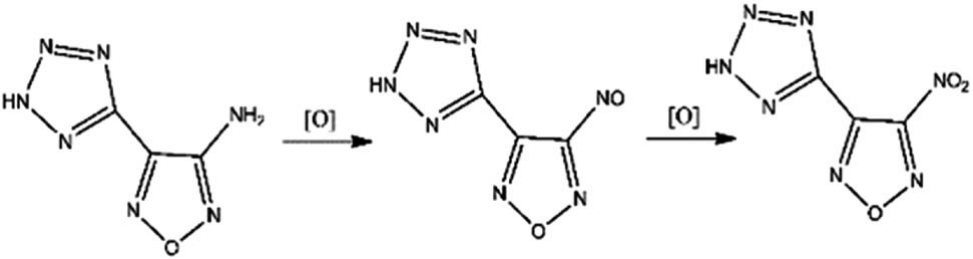
Nitrification process of ATZF.

The oxidizing of H_2_O_2_ is reflected in its own heterogeneous ability. As is shown in Fig. 4, the hydrogen atoms in H_2_O_2_ move to the oxygen atom and undergo isomerization, forming a water molecule with one active oxygen atom. The active oxygen atom is the key to oxidation. The amino-group (–NH_2_) in ATZF is oxidized by H_2_O_2_ to form nitroso-group (–N=O), which is further oxidized by H_2_O_2_ to form a nitro group (Fig. 5).

**Fig. 6:**
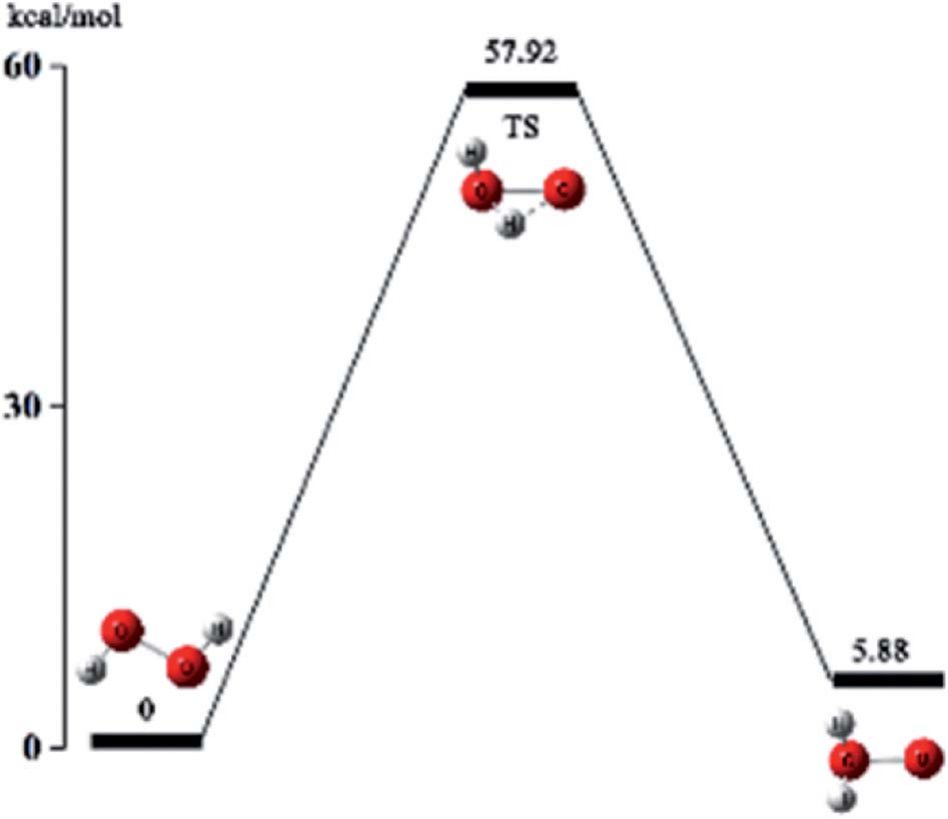
Isomerization of H_2_O_2_.

**Fig. 7:**
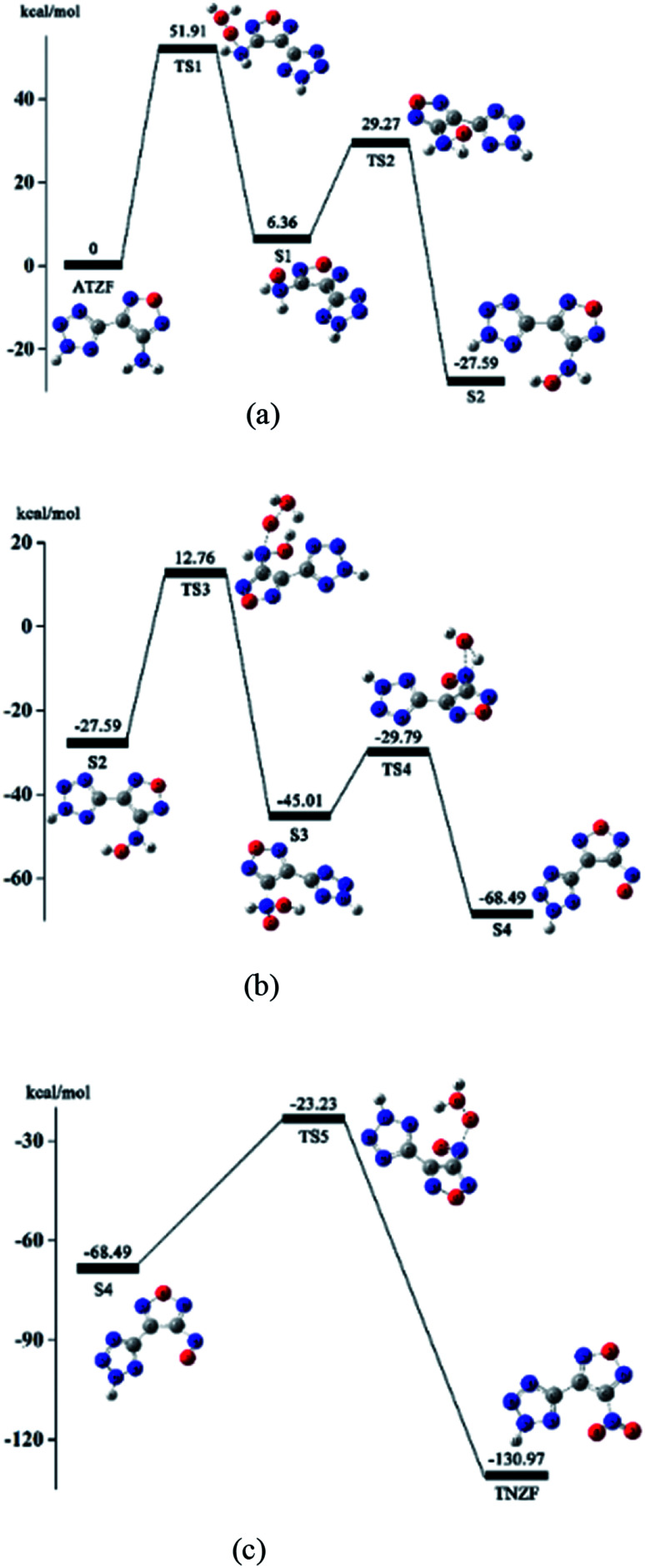
Synthesis of NTZF.

Based on the above conjecture, the Gaussian 09 package was used to explore the transition state of the nitration reaction *via* the TST. The potential energy curves of the H_2_O_2_ isomerization and the nitration reaction are shown in Fig. 6 and 7.

As is shown in Fig. 6, hydrogen atom at one end of H_2_O_2_ molecule break away from the oxygen atom which was bonded to and migrate to another oxygen atom and then reach to the transition state TS. Eventually, the hydrogen atom in H_2_O_2_ completely bonds with the oxygen atom and forms a stable structure of water molecules with an active oxygen atom. The energy of the isomeric H_2_O_2_ molecule is 5.88 kcal mol^−1^ higher than the original H_2_O_2_ molecule. It indicates that the reaction is an endothermic reaction. Therefore, the reaction requires an appropriate temperature and catalyst to activate the H_2_O_2_ molecules.

As Fig. 7(a) shows, the amino group in the ATZF molecule undergoes a nucleophilic attack by the active oxygen, and the reaction reaches the transition state TS1. TS1 is the process of active oxygen dissociating from the binding of water molecules in H_2_O_2_ to the nitrogen atom in the amino group. After the nucleophilic addition of the oxygen atom, the amino group formed a tetrahedral configuration, and the system reached a stable point S1. This is the first oxidation of the H_2_O_2_. Since the new configuration in the stable point S1 structure is not stable enough and the oxygen atom carry a large amount of negative charges, a hydrogen atom on the amino group migrates to the oxygen atom and easily formed a hydroxyl structure. The reaction reached the second transition state TS2. Then, the hydrogen atom detaches from nitrogen atom to bond with the oxygen atom. The system reach the second stable point S2. The energy of the S2 is 27.59 kcal mol^−1^ lower than the ATZF, and the reaction is a heat release process.

Fig. 7(b) shows the hydroxyamino in the molecule of S2 undergoes the second nucleophilic attack by the active oxygen, and reaction reaches the transition state TS3. TS3 is the process of active oxygen dissociating from the binding of water molecules in isomeric H_2_O_2_ to the nitrogen atom in the hydroxyamino. Then, the active oxygen in H_2_O_2_ completely breaks free from the another oxygen atom and forms a stable chemical bond with the nitrogen atom on the hydroxyl amine group. This is the second oxidation of H_2_O_2_. After the nucleophilic addition of the oxygen atom and nitrogen atom, the hydroxyamino formed a new tetrahedral configuration, and the system reached the third stable point S3. Since the hydrogen atom on the imino group is attracted by the charge on the oxygen atom of the hydroxyl and the newly added reactive oxygen species, a hydrogen atom on the imino group can easily migrate to the oxygen atom on the hydroxyl group and forms a water molecule. The reaction reached the transition state TS4. After that, the hydrogen atom on the oxidized imino group is transferred to the oxygen atom of the hydroxyl group to form a water molecule which is detached from the entire molecular system. The system reaches the fourth stable point S4. The energy of the S4 is 68.49 kcal mol^−1^ lower than the ATZF, that is, nitrite reaction will release a lot of heat.

Fig. 7(c) shows the nitroso-group in the molecule of S4 undergoes the third nucleophilic attack by the active oxygen of the isomeric H_2_O_2_, and reaction reaches the transition state TS5. TS5 is the process of active oxygen atom dissociating from the binding of water molecules in isomeric H_2_O_2_ to the nitrogen atom in the nitroso-group. Then, the active oxygen atom detaches from isomeric H_2_O_2_ completely to bond with the nitrogen atom of the nitroso-group to form a more stable Π^3^_4_ system. At this point, the nitration of the amino group is completed and the system reaches the stable point S6 to generate NTZF. The energy of NTZF is 130.97 kcal mol^−1^ lower than the energy of the ATZF, that is, the reaction is a dramatic exothermic reaction.

Through the discussion of the above nitrification mechanism, it was found that the three attacks of active oxygen atom in isomeric H_2_O_2_ is correspond to the three transition states TS1, TS3, and TS5. The three transition states are the key steps affect the reaction rate in the entire nitrification process.

### 3.3 Crystal structure determination

#### 3.3.1 RbNTZF and CsNTZF

**Fig. 8:**
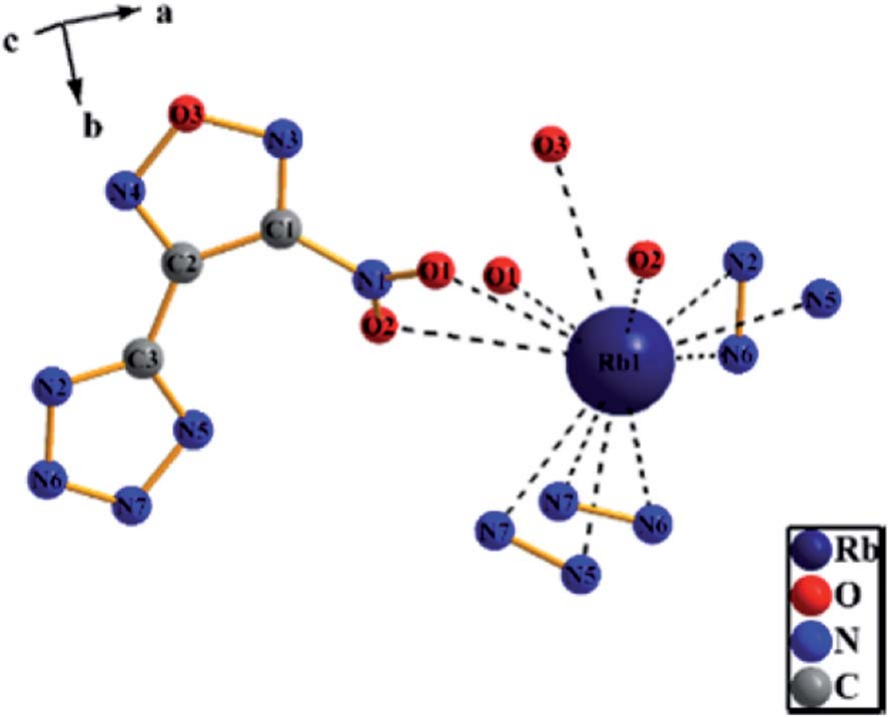
The structure of RbNTZF.

**Fig. 9:**
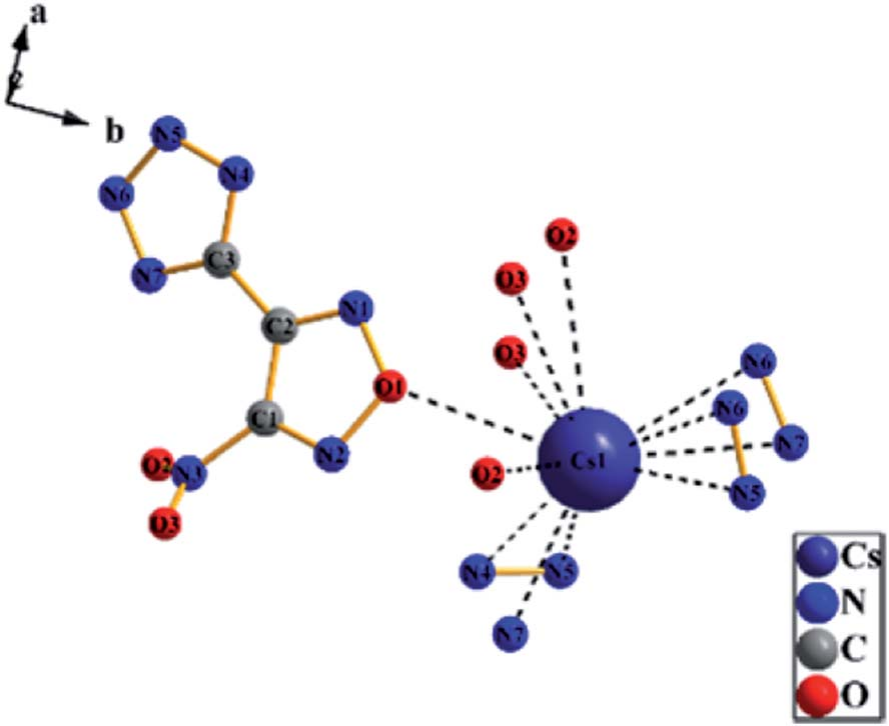
The structure of CsNTZF.

**Fig. 10:**
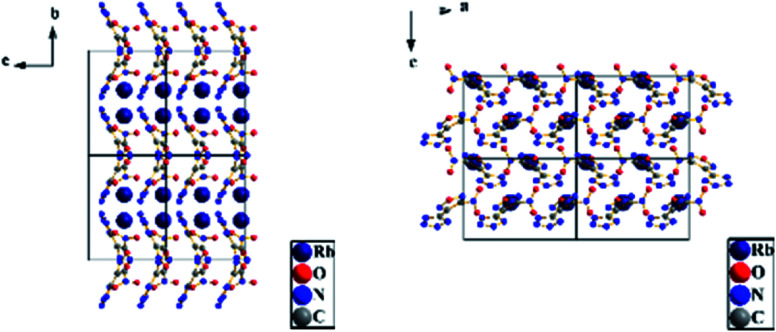
Crystal accumulation diagram of RbNTZF.

**Fig. 11:**
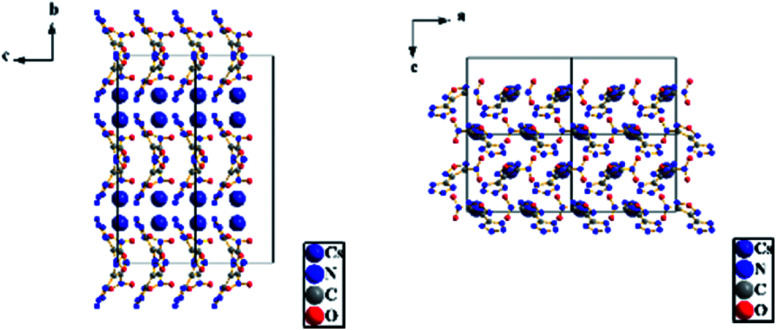
Crystal accumulation diagram of CsNTZF.

A colorless flaky crystal with dimensions of 0.2 × 0.05 × 0.05 mm was chosen for X-ray determination. The related parameter of the crystal are shown in Tables 2 and 3. Fig. 8 and 9 show the structure of ionicsalts. Fig. 10 and 11 show the crystal accumulation diagram.

**Table 2:** Crystal parameter of RbNTZF

Identification code	RbNTZF
Empirical formula	C_3_N_7_O_3_Rb
Formula weight	267.57
Temperature/K	153.15
Crystal system	Orthorhombic
Space group	*Pca*2_1_
*a*/Å	10.203(2)
*b*/Å	9.936(2)
*c*/Å	7.3949(15)
*α*/°	90
*β*/°	90
*γ*/°	90
Volume/Å^3^	749.7(3)
*Z*	4
*ρ*_calc_/g cm^−3^	2.37
*μ*/mm^−1^	6.599
*F*(000)	512.0
Crystal size/mm^3^	0.2 × 0.05 × 0.05
Radiation	MoKα (*λ* = 0.71073)
2*θ* range for data collection/°	4.1 to 54.958
Index ranges	−13 ≤ *h* ≤ 7, −11 ≤ *k* ≤ 12, −9 ≤ *l* ≤ 9
Reflections collected	2854
Independent reflections	1601 [*R*_int_ = 0.0283, *R*_sigma_ = 0.0387]
Data/restraints/parameters	1601/1/127
Goodness-of-fit on *F*^2^	1.253
Final *R* indexes [*I* ≥ 2*σ*(*I*)]	*R*_1_ = 0.0315, w*R*_2_ = 0.0923
Final *R* indexes [all data]	*R*_1_ = 0.0350, w*R*_2_ = 0.1127

**Table 3:** Crystal parameter of CsNTZF

Identification code	CsNTZF
Empirical formula	C_3_N_7_O_3_Cs
Formula weight	315.01
Temperature/K	153.15
Crystal system	Orthorhombic
Space group	*Pca*2_1_
*a*/Å	10.376(2)
*b*/Å	10.128(2)
*c*/Å	7.6675(15)
*α*/°	90
*β*/°	90
*γ*/°	90
Volume/Å^3^	805.8(3)
*Z*	4
*ρ*_calc_/g cm^−3^	2.60
*μ*/mm^−1^	4.592
*F*(000)	584.0
Crystal size/mm^3^	0.12 × 0.08 × 0.04
Radiation	MoKα (*λ* = 0.71073)
2*θ* range for data collection/°	4.022 to 54.946
Index ranges	−13 ≤ *h* ≤ 13, −13 ≤ *k* ≤ 7, −6 ≤ *l* ≤ 9
Reflections collected	3059
Independent reflections	1502 [*R*_int_ = 0.0318, *R*_sigma_ = 0.0366]
Data/restraints/parameters	1502/7/127
Goodness-of-fit on *F*^2^	1.273
Final *R* indexes [*I* ≥ 2*σ*(*I*)]	*R*_1_ = 0.0301, w*R*_2_ = 0.0921
Final *R* indexes [all data]	*R*_1_ = 0.0388, w*R*_2_ = 0.1434

### 3.4 Stability of NTZF

#### 3.4.1 Thermal analysis

**Fig. 12:**
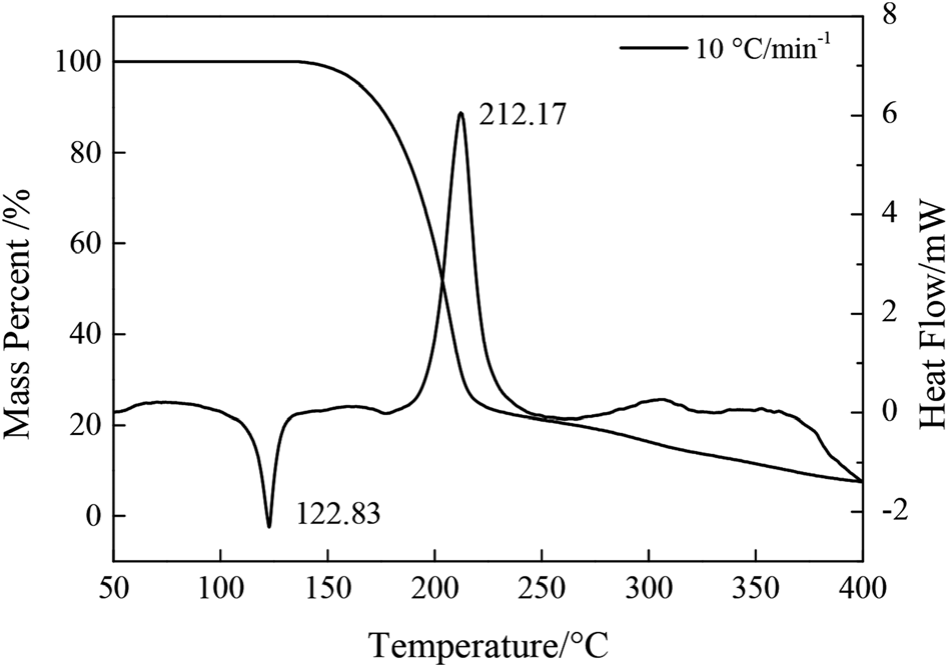
TG-DSC curves of NTZF with the heating rate 10 °C min^−1^.

The TG-DSC curves of NTZF with a heating rate of 10 °C min^−1^ is shown in Fig. 12. In the DSC curve, an endothermic peak appears at 122.83 °C, while the TG curve remains unchanged, the reason is that NTZF undergoes a phase change and endothermically melts. The curve's melting range is short and the peak is sharp, it indicates that the product is pure. In the TG curve, there is a sharply mass loss platform, the mass loss rate is 81.36%, corresponding to the exothermic decomposition peak temperature of the DSC curve at 176.83–248.50 °C. The residual solid is assumed to be the carbon element in the molecule (19.68% of theoretical value). Subsequently, the carbon atoms oxidize with excess oxygen atoms again, so the TG curve is still slowly declining. For NTZF, the energy release has been completed at 248.50 °C. It indicates that NTZF is an ideal energetic compound with a high concentration of energy release.

**Fig. 13:**
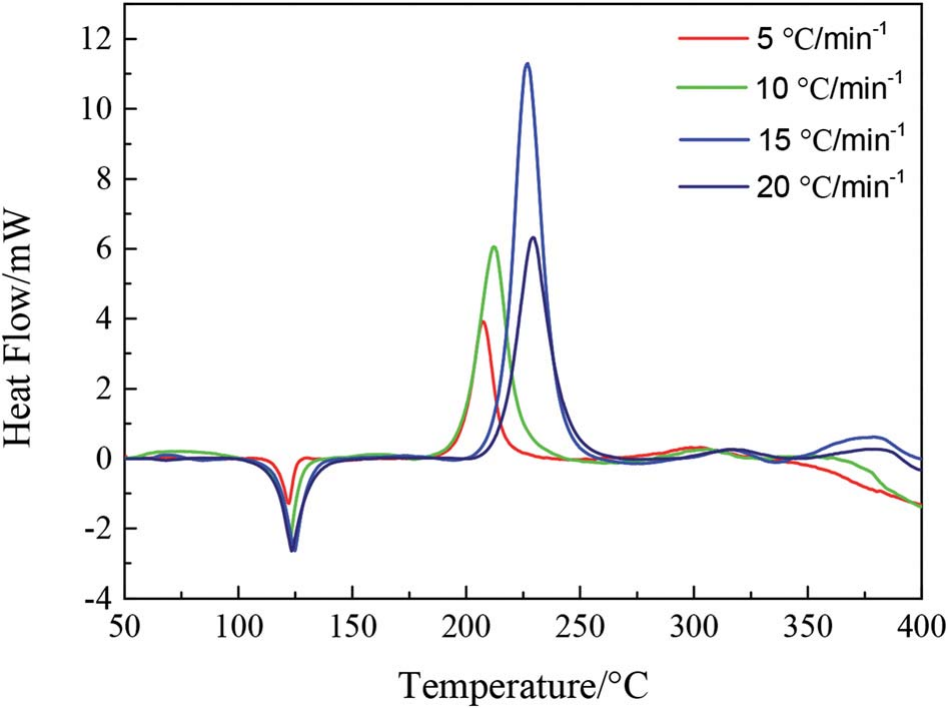
DSC curves of NTZF with the heat rate 5, 10, 15, and 20 °C min^−1^.

DSC curves of NTZF with heating rate 5, 10, 15, and 20 °C min^−1^ are shown in Fig. 13. The kinetic data of NTZF thermal decomposition were calculated by Kissinger method (eqn (1)) and Ozawa method (eqn (2)) respectively. The results are shown in Table 4.

**Table 4:** Thermal decomposition kinetics data of NTZF

*β*/°C min^−1^	Peak temperature *T*_p_/°C	Kissinger			Ozawa	
		*E*/ kJ mol^−1^	lg *A*/s^−1^	*R*	*E*/ kJ mol^−1^	*R*
5	207.58	100.20	8.60	0.94	103.10	0.95
10	212.83					
15	227.00					
20	229.33					

Kissinger equation^15^:1
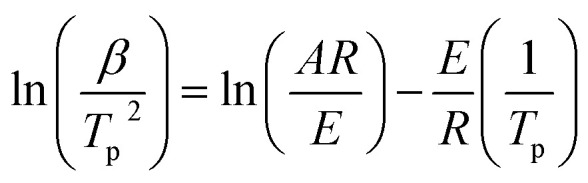


Ozawa equation^16^:2
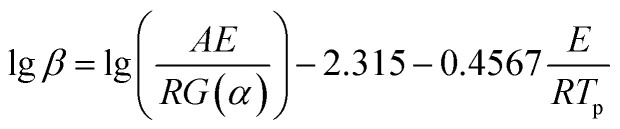
where *β* is the heating rate [K min^−1^], *T*_p_ is the peak temperature [K], *E* is the activation energy [KJ mol^−1^], *A* is the pre-exponential factor [s^−1^], and *R* is the gas constant [J min^−1^ K^−1^].

#### 3.4.2 Sensitivity tests

Table 5 shows the sensitivity data of NTZF. It indicates that NTZF is insensitive toward impact and friction, which are all higher than TNT (impact, 15 J; friction, 353 N) and has a certain ignition performance.

**Table 5:** Sensitivity data of NTZF

Impact/J	Friction/N	Flame/cm
>40	>360	22.50

#### 3.4.3 Detonation pressure and detonation velocity

The relevant electron energy parameters of NTZF are obtained by analyzing the optimized NTZF geometry results at the B3LYP/6-311 ++ G ** level (Table 6). By substituting the parameters into eqn (3) and (4),^17^ the standard molar formation enthalpy of NTZF at 298 K can be calculated. Then, the standard molar enthalpy of formation is introduced into eqn (5)–(7).^18,19^ The theoretical detonation velocity and detonation pressure of NTZF can be predicted by the semi-empirical K–J equation. The formation enthalpy, detonation velocity and detonation pressure are listed in Table 7. The calculated detonation velocity and detonation pressure are both higher than TNT (*D* = 6881 m s^−1^, *P* = 19.5 GPa).3
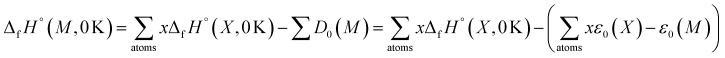
4
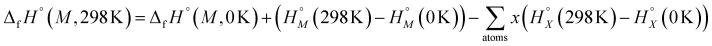

5 *P* = *Kρ*^2^*φ*

6 *D* = *Aφ*^1/2^(1 + *Bρ*)

7
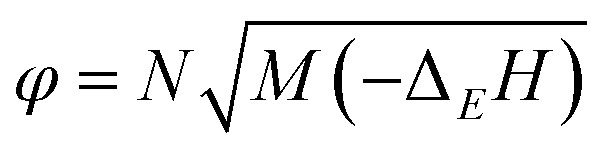


**Table 6:** Relevant electron energy parameters of NTZF

*ε*_0_ + *ε*_ZPE_ [a.u.]	*ε*_ZPE_ [a.u.]	*H*_corr_ [a.u.]
−723.71	0.07	0.09

**Table 7:** Detonation data of NTZF

Δ_f_*H*° (298 K) [kJ mol^−1^]	*D* [m s^−1^]	*P* [GPa]
256.32	7838.17	27.32

## 4. Conclusions

(1) Based on the density functional theory (DFT), the molecular structure of NTZF is designed and optimized. The synthesis mechanism of NTZF is explored by the transition state theory for the first time.

(2) NTZF was successfully synthesized based on the theoretical design. Its ionicsalts (RbNTZF and CsNTZF) were synthesized and studied by single-crystal X-ray diffraction firstly.

(3) The stability and detonation performance of NTZF were studied. It has good thermal stability (the exothermic decomposition peak temperature is at 176.83–248.50 °C). NTZF is insensitive toward impact and friction (impact > 40 J, friction > 360 J). The formation heat, detonation velocity and detonation pressure is 256.32 kJ mol^−1^, 7.838 km s^−1^ and 27.32 GPa.

These indicate that NTZF is an energetic heterocyclic compound with low sensitivity which has broad application prospects in low explosives and gas generant.

## Conflicts of interest

There are no conflicts to declare.

## Supplementary Material

RA-008-C8RA02682C-s001

RA-008-C8RA02682C-s002
