# Supplementary material for: 3-Nitro-4-(tetrazol-5-yl) furazan: theoretical calculations, synthesis and performance
Source: RSC Adv. 2018 Apr 18;8(26):14589–96. doi: 10.1039/c8ra02682c (PMC9079957; doi:10.1039/c8ra02682c)
Supplement: RA-008-C8RA02682C-s001 [file RA-008-C8RA02682C-s001.pdf]

Electronic Supplementary Material (ESI) for RSC Advances.
This journal is © The Royal Society of Chemistry 2018

Supplementary material for: 3-nitro-4-(tetrazol-5-yl) furazan: Theoretical calculation, Synthesis and Performance

Zhiyue Han[1*], Qi Jiang, Zhiming Du, Yupeng Zhang, Yuezhen Yang

State Key Laboratory of Explosion Science and Technology, Beijing Institute of Technology, 5 South Zhongguancun Street, Haidian District, Beijing, 100081, China

The structure of NTZF was optimized with B3LYP / 6-311 ++ G ** [13] using the Gaussian 09 software package. The geometric structure, bond lengths and bond angles of the NTZF molecule are shown in supplementary Table 1 and Table 2.

Supplementary Table 1 Bond length of NTZF

parameter	Bond lengths /Å	parameter	bond lengths /Å
O1—N5	1.36	N7—N9	1.32
O1—N6	1.37	N7—C11	1.33
C2—N6	1.31	N8—N10	1.30
C3—N5	1.30	N8—C11	1.36
C2—C3	1.43	N9—N10	1.33
C2—C11	1.46	N12—O13	1.22
C3—N12	1.47	N12—O14	1.21
H4—N9	1.01		

Supplementary Table 2 Bond angles of NTZF

bond	bond angles / °	bond	bond angles / °
N5—O1—N6	112.03	N9—N7—C11	100.85
C3—C2—N6	107.02	N10—N8—C11	106.04
C3—C2—C11	130.05	N8—N10—N9	105.88
C2—C3—N5	110.36	N7—N9—N10	114.62
C2—C3—N12	129.07	C2—C11—N7	122.77
O1—N5—C3	104.64	N7—C11—N8	112.60
O1—N6—C2	105.94	C3—N12—O13	115.27

* Corresponding author. Tel.: +86-01-68912765. Fax: +86-01-68912765

e-mail: hanzhiyue@bit.edu.cn (Zhiyue Han)

H4—N9—N7	122.90	O13—N12—O14	117.41
